# Application and Progress of Loading Strategies in Bone Tissue Engineering Scaffolds for Bone Regeneration

**DOI:** 10.3390/bioengineering12121336

**Published:** 2025-12-08

**Authors:** Tenglong Luo, Zhangfeng Huang, Chen Fu, Jiecong Wang

**Affiliations:** 1 Department of Plastic Surgery, Union Hospital, Tongji Medical College, Huazhong University of Science and Technology, Wuhan 430022, China; m202376239@hust.edu.cn (T.L.);; 2 Department of Thoracic Surgery, Union Hospital, Tongji Medical College, Huazhong University of Science and Technology, Wuhan 430022, China

**Keywords:** craniofacial bone defects, loading strategies, bone tissue engineering scaffolds, bone regeneration

## Abstract

Craniofacial bone defects of critical size, caused by trauma, tumors, infections, or congenital maldevelopment, represent a major challenge in plastic and reconstructive surgery. Autologous bone grafting is considered the gold standard, but limitations such as donor site morbidity and limited availability have prompted the development of artificial bone tissue engineering scaffolds. In recent years, bioactive scaffolds have been increasingly utilized in favor of inert biomaterials due to their immunomodulation and osteoinduction capabilities. This review methodically summarizes loading strategies for the functionalization of scaffolds with bioactive components, including cell regulatory factors, drugs, ions, stem cells, exosomes, and components derived from human tissues or cells to promote bone regeneration. The following mechanisms are involved: (1) the polarization of macrophages (M1-M2 transition), (2) the dynamic regulation of bone metabolism, and (3) the coupling of osteogenesis and angiogenesis. This review focuses on innovative delivery systems, such as 3D-printed scaffolds, nanocomposites and so on, that enable spatiotemporal control of bioactive cargo release. These address key challenges, such as infection resistance, vascularization, and mechanical stability in the process of bone regeneration. In addition, the article discusses emerging technologies, including stem cells and exosome-based acellular therapies, which demonstrate potential for personalized bone regeneration. This review integrates immunology, materials science, and clinical needs, providing a roadmap for the design of next-generation bone tissue engineering scaffolds to overcome critical-sized bone defects.

## 1. Introduction

Irregular craniofacial bone defects may be caused by trauma, tumors, infections, and congenital developmental defects [1,2]. Craniofacial bone defects that exceed critical size and cannot heal spontaneously remain a major challenge in plastic and reconstructive surgery [3]. The craniofacial region is composed of 23 bones, the critical size defects of these bones may cause severe craniofacial dysfunction and affect the normal craniofacial structure [4]. Bone defect regeneration is crucial for restoring craniofacial function and facial aesthetics. Treatment methods include autologous bone transplantation, allograft bone transplantation, induced membrane techniques, distraction osteogenesis, and bone tissue engineering scaffold implantation. Among these, autologous bone transplantation remains the gold standard for the treatment of bone defects [5]. However, autologous bone grafting presents several limitations in clinical practice, such as donor site damage, limited availability of bone tissue, and difficulty in meeting the complex geometric requirements of craniofacial bone defects [6,7].

Advances in biomaterials science have led to the emergence of bone tissue engineering scaffolds, presenting new avenues for the treatment of large craniofacial bone defects [8,9]. Bone tissue engineering scaffolds are constructed and cultured using bone tissue engineering techniques, thereby enabling large-scale and personalized production according to clinical needs. Such techniques have advantages such as low cost and ease of acquisition. Early bone tissue engineering scaffolds primarily aimed to achieve stable physicochemical properties and good biocompatibility. However, following research into the complex mechanisms of bone regeneration and progress in bone immunology-related studies, an increasing number of researchers have studied materials with bioactive functions [10,11]. The latter approach can induce bone regeneration and promote in situ repair of bone defects.

Previous studies have identified numerous bioactive substances capable of promoting bone regeneration, including cell regulatory factors, pharmaceutical agents, bioactive metal ions, stem cells, and active components derived from human tissues or cells. These substances facilitate bone regeneration through multiple mechanisms, including the modulation of local immune microenvironments, the regulation of bone cell activities, promoting vascularization, and inhibiting bacterial growth. The functionalization of bone tissue engineering scaffolds by incorporating and controlling the release of these bioactive substances has emerged as a promising strategy. In recent years, researchers have developed various scaffold designs incorporating these active substances, aiming to achieve post-implantation biological regulation and enhanced bone regeneration. These experimental approaches have yielded encouraging results, demonstrating considerable potential for clinical translation.

Therefore, this article provides a comprehensive overview of the application of loading strategies in bone tissue engineering scaffolds for biological regulation in bone regeneration, offering new insights and methodologies for researchers in this field.

## 2. Bone Regeneration and Related Mechanisms

**Figure 1:**
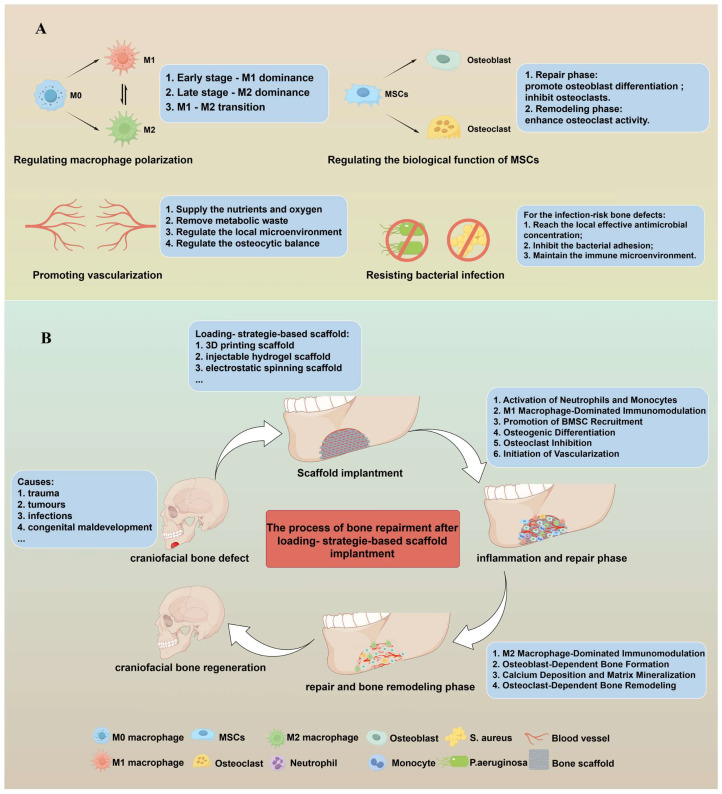
The process and related mechanisms of craniofacial bone regeneration after loading-strategy-based scaffold implantation. (**A**) These mechanisms of ideal craniofacial bone regeneration include modulating local inflammation by promoting macrophage M2 polarization, enhancing osteogenic capacity through promoted vascularization, and regulating the osteoblast differentiation of MSCs as well as MSCs’ modulatory effects on osteoclasts at various stages of bone regeneration, mitigating post-implantation infection risk via antibacterial activity. (**B**) The process of craniofacial bone regeneration after loading-strategy-based scaffold implantation, and the dynamic changes in macrophage phenotype, osteocytes, vascularization and so on during bone repair processes.

During the process of bone regeneration, immune cells and cytokines play a regulatory role in bone homeostasis and the regenerative process. In addition, bone cells have been found to regulate the biological functions of immune cells [12,13]. Research on related immune regulatory networks revealed that macrophages participate in bone regeneration. In bone defects, a delicate dynamic balance among osteogenesis-related cells, including osteoblasts, osteoclasts, and mesenchymal stem cells, must be maintained to maximize bone regeneration [14]. Furthermore, early angiogenesis and the subsequent formation of a mature vascular network have been demonstrated to directly influence the success or failure of bone defect repair [15]. Figure 1 displays the process and related mechanisms of craniofacial bone regeneration after loading-strategy-based scaffold implantation.

### 2.1. Bone Regeneration-Macrophages

Extensive research in osteoimmunology has shown that bone regeneration is mediated by a complex immune network. Notably, macrophages play a pivotal role and are widely recognized as central regulators of tissue regeneration [16]. Under physiological conditions, tissue-resident macrophages in bone contribute to skeletal and bone marrow homeostasis by supporting the functional activities of osteoblasts [17]. In inflammatory environments, macrophages act as phagocytes, serving a dual role. They effectively prevent pathogen invasion, clear necrotic tissue and cellular debris, and modulate inflammatory responses; moreover, macrophages differentiate into osteoclasts under specific conditions [12,13,18]. In bone remodeling, macrophages and the factors they secrete (e.g., MMP, VEGF) play a pivotal role in the transformation of soft and hard callus tissue, vascularization, and bone deposition [17,19].

During bone regeneration following defect formation, macrophages can polarize into either the pro-inflammatory M1 or anti-inflammatory M2 phenotypes and can transition between these states depending on microenvironmental cues [17,20]. The regenerative process is characterized by an initial M1-dominant phase that gradually shifts to an M2-dominant phase. In early stages, infiltrating macrophages at the defect site primarily exhibit M1 polarization. During repair, these macrophages secrete bioactive cytokines that promote neutrophil apoptosis, thereby driving their own phenotypic transition from M1 to M2. The M2 macrophages then phagocytose apoptotic neutrophils, alleviating local inflammation in the damaged tissue [21].

### 2.2. Bone Regeneration—Osteocytes

Under physiological conditions, bone tissue maintains a dynamic equilibrium known as bone remodeling. The osteoblasts and osteoclasts are the osteocytes primarily involved in this process. In summary, osteoclast differentiation is initiated by the recruitment of these cells, which produce acidic substances and enzymes that dissolve the bone matrix, leading to bone resorption and apoptosis. Subsequently, osteoblast differentiation is initiated, and these cells migrate towards areas of bone matrix resorption and synthesize new bone tissue and regulate its mineralization, ultimately undergoing apoptosis or differentiation into osteocytes [22].

The process of bone regeneration following bone defects is induced by local cytokines, chemokines, and other factors, which in turn stimulate the recruitment of mesenchymal stem cells. The promotion of osteogenic differentiation and the inhibition of osteoclast activity are driven by specific mechanisms, facilitating bone tissue regeneration [14]. In bone defect treatments involving scaffold implantation, most bone tissue engineering scaffolds must support the attachment and proliferation of osteoblasts and mesenchymal progenitor cells, as bone conductors of the scaffold structure. Subsequently, the production of extracellular organic bone matrix by osteoblasts, in conjunction with inorganic salt deposition, plays an instrumental role in the process of bone regeneration, thereby facilitating bone healing. The osteocytes implicated in this process predominantly consist of osteoblasts and osteoclasts [23,24].

### 2.3. Bone Regeneration—Vascularization

Bone tissue is a highly vascularized tissue that relies heavily on the supply of vascular branches during its growth and development [25]. Notably, vascularization supports bone regeneration, and dysfunctional or delayed vascularization is a major obstacle to bone tissue regeneration. The close spatial and temporal relationship between osteogenesis and angiogenesis is referred to as “angiogenesis–osteogenesis coupling” [26]. In the tissue repair of large bone defects, angiogenesis is required to supply the nutrients and oxygen supporting the repair of the defect site and to remove waste products generated during cellular metabolism. Furthermore, vascularization also provides a suitable microenvironment for bone regeneration and reconstruction [15]. Following the implantation of bone tissue scaffolds into the body, rapid vascularization within the scaffold promotes the migration of regenerating bone tissue toward the scaffold center. For bone tissue scaffolds loaded with cells, timely vascularization is necessary to support cell migration, growth, and viability within the scaffold, as well as to enable its bone-inductive effects [27]. Therefore, effective healing of critical-sized bone defects depends heavily on achieving adequate vascularization, which remains a major challenge in the development of bone tissue engineering scaffolds.

### 2.4. Interaction Among the Mechanisms

The aforementioned mechanisms do not operate in isolation but rather interact in a complex manner. For instance, recent studies suggest that M1 macrophages transiently promote angiogenesis in the early inflammatory phase by secreting factors such as TNF-α. Subsequently, the polarization to the M2 phenotype, characterized by CD206^+^ expression, stabilizes the nascent vessels and angiogenesis-osteogenesis coupling [28,29]. Furthermore, the newly formed blood vessels directly activate osteogenic signaling pathways in mesenchymal stem cells (MSCs) via factors like BMP-2 and PDGF secreted by endothelial cells, thereby promoting bone formation [30,31,32]. In addition, upon undergoing osteogenic differentiation, MSCs secrete immunomodulatory factors, including TSG-6 and PGE2, which further promote the polarization of macrophages toward the M2 phenotype, establishing a positive feedback loop [33,34].

Previous studies have investigated the intricate mechanisms of bone immune regulation, the equilibrium among osteogenic cells, and the vascularization processes involved in bone regeneration. Researchers have devised a range of bone tissue engineering scaffolds that possess pertinent biological regulatory functions by loading diverse bioactive components, such as cell regulatory factors, pharmaceutical agents, bioactive metal ions, stem cells, and active components derived from human tissues or cells into the scaffolds. These loading strategies have been designed to enhance bone regeneration and promote the therapeutic effects of bone defects.

## 3. Design Principles and Applications of Bone Tissue Engineering Scaffolds Based on Loading Strategies

**Figure 2:**
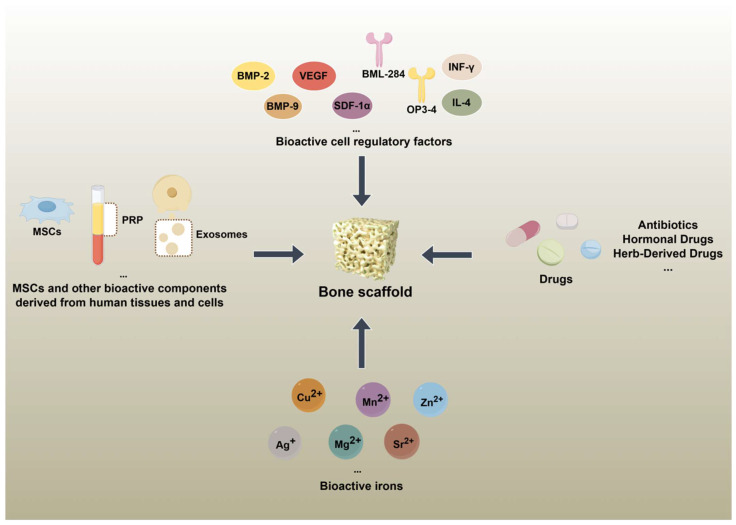
The design principles and applications of bone tissue engineering scaffolds based on loading strategies. MSCs: Marrow stromal cells; PRP: Platelet-rich plasma; BMP-2: Bone morphogenetic protein 2; BMP-9: Bone morphogenetic protein 9; VEGF: Vascular endothelial growth factor; SDF-1α: Stromal cell-derived actor 1 alpha; IFN-γ: Interferon-gamma; IL-4: Interleukin-4.

In the early stages of bone tissue engineering scaffold design and manufacturing, researchers primarily explored properties such as biocompatibility, mechanical performance, structural biomimicry, and degradation rate. These studies aimed to develop new scaffold platforms for bone regeneration by leveraging appropriate physical and chemical properties, while neglecting the potential of the scaffold itself to serve as a local biological regulator. Subsequently, bone tissue engineering scaffolds were endowed with inherent biological regulatory capabilities via various strategies. Notably, the loading strategy—incorporating bioactive substances with regulatory properties into the scaffold—has yielded some successful outcomes. Figure 2 displays the design principles and applications of bone tissue engineering scaffolds based on loading strategies.

### 3.1. Loading Bioactive Cell Regulatory Factors into Scaffolds

During bone regeneration, a significant number of cell regulatory factors and complex signaling networks present within the local immune microenvironment assume a regulatory role [35]. Consequently, bone regeneration can be induced by directly loading cell regulatory factors or regulating related cell signaling pathways by loading signaling pathway modulators.

#### 3.1.1. Loading of Bone Morphogenetic Protein 2

Bone morphogenetic protein-2 (BMP-2) exhibits potent osteoinductive properties and immunomodulatory effects. Studies have demonstrated that BMP-2 can directly promote osteogenic differentiation while inhibiting osteoclast activity. Moreover, the protein can induce macrophage recruitment and modulate their polarization, thereby regulating the local immune microenvironment [36,37]. However, the rapid release of BMP-2 at the target site may lead to adverse effects such as ectopic ossification and irregular bone formation [38].

To mitigate these undesirable effects, BMP-2 was encapsulated within bone tissue scaffolds, and spatiotemporal controlled delivery strategies can be employed to significantly enhance the efficacy of bone regeneration. For instance, Jixing Ye et al. [39] incorporated BMP-2 into a hydrogel-based bone scaffold composed of polyethylene glycol (PEG), gelatin methacrylate (GelMA), and demineralized bone matrix (DBM), thereby enabling sustained BMP-2 release and promoting osteogenic differentiation. Ultimately, a rat critical-sized calvarial defect model showed successful repair. Similarly, Seoyun Lee et al. [40] utilized microsphere encapsulation technology to load BMP-2-loaded alginate microspheres into a bone tissue engineering scaffold, achieving prolonged BMP-2 release over 28 days, which significantly enhanced bone regeneration in large bone defects.

Furthermore, some studies have loaded BMP-9 or BMP-2 substitutes, such as P28, into scaffolds and confirmed similar bone immunomodulatory effects to BMP-2-loaded scaffolds [41,42].

#### 3.1.2. Loading of Angiogenic Regulators

Vascular endothelial growth factor (VEGF) is a key regulator of angiogenesis, which has been incorporated into bone tissue engineering scaffolds to leverage its biological regulatory effects post-implantation. For example, Xu Chen et al. [43] employed an adsorption method to load VEGF into a heparin-gelatin-hydroxyapatite-tricalcium phosphate (HG-HA-TCP) scaffold, achieving controlled VEGF release after in vivo implantation. The sustained VEGF delivery upregulated the expression of BMP-2, CD31, RUNX2, and vWF, thereby enhancing osteogenesis-angiogenesis coupling and establishing a favorable osteogenic microenvironment to accelerate bone regeneration. The therapeutic efficacy of VEGF-loaded scaffolds has been corroborated by additional research, demonstrating that rapid VEGF release in the early post-implantation phase initiates vascularization, ensuring adequate blood supply for subsequent osteogenic processes [44].

#### 3.1.3. Loading of Cellular Signaling Pathway Modulators

The incorporation of bone regeneration-related signaling pathway modulators into scaffolds can exert positive regulatory effects on osteogenesis by activating specific signaling cascades. For instance, Minhao Wu et al. [45] utilized biomimetic dopamine chemistry and self-assembly techniques to load BML-284 (BML), a potent and highly selective Wnt signaling activator, onto a multilayered bone tissue engineering scaffold. The sustained release of BML from the scaffold surface significantly enhanced osteogenic differentiation and angiogenesis, while also stimulating M2 macrophage polarization. This approach facilitated the recruitment of endogenous stem cells and endothelial cells to the injury site, creating a favorable regenerative microenvironment while simultaneously suppressing osteoclastogenesis, thereby promoting bone healing.

In another study, Cangyou Xie et al. [46] utilized an impregnation method to load OP3-4, a receptor activator of NF-κB ligand (RANKL)-binding peptide, into a nanogel-based scaffold. Their findings revealed that this signaling pathway modulator could potentiate BMP-2 signaling and upregulate osteogenic gene expression (e.g., Smad-Runx2 axis), leading to enhanced osteoblast differentiation and bone regeneration. This strategy effectively repaired critical-sized calvarial defects in a murine model.

#### 3.1.4. Loading of Multiple Bioactive Cell Regulatory Factors

Research has advanced our understanding of the complex, multi-stage regulatory networks involved in bone regeneration. Consequently, recent developments in single-factor-loaded bone tissue scaffolds have led to the design of scaffolds incorporating dual or multiple cellular regulatory factors. Through sophisticated loading strategies and hierarchical scaffold design, these systems enable the sequential release of distinct biological factors, thereby achieving stage-specific modulation to enhance bone regeneration and repair efficacy.

Jiawei Wei et al. [47] fabricated a PLGA-based bone scaffold embedded with hydroxyapatite (HA) microspheres encapsulating bone morphogenetic protein-2 (BMP-2) via 3D printing technology, followed by surface immobilization of stromal cell-derived factor-1α (SDF-1α). This innovative design achieved both spatial and temporal control over factor delivery. The rapid release of surface-bound SDF-1α effectively recruited bone marrow-derived mesenchymal stem cells (BMSCs) to the defect site; subsequently, the sustained release of BMP-2 from HA microspheres promoted osteogenic differentiation of the recruited BMSCs. At 14 weeks post-implantation in the rat calvarial defect model, the scaffold demonstrated a larger volume of bone regeneration and sustained osteogenic mineralization capacity compared to the control scaffold. In a similar manner, Kara L. Spiller et al. [48] developed a bone regeneration scaffold capable of early transient release of interferon-γ and subsequent sustained release of interleukin-4. This system induced rapid macrophage M1 polarization in the early stage and achieved sustained M2 polarization, thereby effectively improving vascularization levels and promoting bone regeneration.

### 3.2. Loading Drugs into Scaffolds

Bone tissue engineering scaffolds can achieve controlled drug release through physical encapsulation, nanotechnology, and other methodologies, regulating the local osteoimmune microenvironment. This approach enables multiple biological functions, including infection prevention, modulation of local inflammatory responses, and promotion of osteogenic differentiation.

#### 3.2.1. Loading of Antibiotic

Bone defects are often accompanied by local bacterial infections or potential infection risks. Pathogenic bacteria can significantly impair local tissue integrity and osteogenic capacity, leading to vascular damage and osteonecrosis. Moreover, systemic antibiotic administration frequently fails to achieve effective drug concentrations at the infection site, resulting in delayed bone healing, nonunion, or even amputation. Therefore, incorporating antibiotics into bone tissue scaffolds with controlled spatiotemporal release kinetics may provide superior local anti-infection efficacy, effectively maintaining a favorable immune microenvironment and facilitating bone regeneration.

For instance, Jianyi Li et al. [49] utilized a dual-nozzle 3D printing technique to uniformly load vancomycin into the pores of a nano-hydroxyapatite/polylactic acid (nHA-PLA) scaffold. This approach enabled sustained vancomycin release, demonstrating excellent antibacterial efficacy against Staphylococcus aureus while effectively combating local infection and promoting bone regeneration. In other similar studies, researchers employed microsphere technology or composite nanoparticle technology to load penicillin, minocycline, and other antibiotics onto bone tissue engineering scaffolds. These studies also demonstrated that this loading strategy can achieve effective antibacterial effects, prevent graft infections, and create a favorable local immune microenvironment for subsequent bone regeneration [50,51].

#### 3.2.2. Loading of Hormonal Drugs

Various natural and synthetic hormones have been demonstrated to play crucial roles in bone metabolism, with dexamethasone (Dex) and parathyroid hormone (PTH) being two classical representatives.

Dexamethasone (Dex), a synthetic glucocorticoid, is widely utilized for its anti-inflammatory and osteogenic properties. However, high local concentrations of Dex may induce cytotoxic effects, necessitating optimized loading strategies to achieve sustained local release in bone tissue engineering scaffolds. For instance, Sheng Yang et al. [52] developed a scaffold by electrostatically adsorbing Dex onto mesoporous silica nanoparticles, followed by surface sealing with polydopamine and scaffold fabrication via selective laser sintering (SLS). This system achieved delayed and sustained Dex release, effectively stimulating osteogenic differentiation of BMSCs and promoting bone regeneration. Co-loading Dex and BMP-2 into bone tissue engineering scaffolds has been shown to produce a synergistic effect, with Dex stabilizing the secondary structure of BMP-2 and promoting the recognition of BMP-2 with its surface receptors [53,54].

Parathyroid hormone (PTH), an FDA-approved anabolic agent, exhibits dual osteogenic, osteoclastic, and angiogenic functions. Successful application of PTH for localized bone defect repair requires intricate carrier-mediated loading and delivery. Shi Li et al. [55] engineered a near-infrared (NIR)-activatable scaffold with dual-mode release kinetics for PTHrP-2 (a PTH derivative), demonstrating NIR-triggered pulsatile release alongside long-term sustained release. This system significantly enhanced BMSC proliferation and osteogenic differentiation, while also promoting HUVEC proliferation, migration, and tube formation, thereby facilitating vascularization in vitro. The scaffold achieved balanced osteogenesis/osteoclast activity and enhanced vascularization, resulting in superior bone regeneration efficacy.

#### 3.2.3. Loading of Herb-Derived Small Molecule Drugs

In traditional medicine, various herbal extracts are used for treating bone-related disorders such as fractures. Modern pharmacology has derived the osteoinductive small-molecule compounds found in these medicinal plants, demonstrating their significant potential for incorporation into bone tissue engineering scaffolds.

Inspired by the traditional use of Drynaria fortunei (Gusuibu) in fracture treatment as documented in Compendium of Materia Medica, Haixiong Lin et al. [56] incorporated total flavonoids from Drynaria rhizome (TFRD)—the primary active constituents of this herb—into a composite scaffold. The sustained TFRD release enhanced osteogenic gene/protein expression and mineralized matrix deposition in BMSCs. Mechanistically, TFRD activated the PI3K/AKT/HIF-1α pathway, simultaneously promoting osteogenesis and angiogenesis. This scaffold effectively reduced infection, enhanced vascularization, and accelerated bone regeneration in a rat segmental bone defect model.

Epimedium (Yinyanghuo), a traditional Chinese herb for osteoporosis treatment, produces icariin (ICA) as its primary enzymatic hydrolysate. Yanbing Liu et al. [57] developed an ICA-loaded scaffold and revealed that sustained ICA release upregulated ALP activity and ECM mineralization by modulating the Runx2, OPN, and OCN pathways, ultimately enhancing bone regeneration. Another study employed icaritin (the hydrolyzed derivative of ICA), demonstrating that its controlled release effectively promoted MSC migration and osteogenic differentiation through the Integrin-FAK-ERK1/2-Runx2 axis, facilitating bone repair [58].

Additionally, multiple plant-derived bioactive molecules, including baicalin, berberine (BBR), gymnemic acid, and naringin, have been successfully incorporated into bone scaffolds. These compounds exhibit multifunctional therapeutic effects through antimicrobial/anti-inflammatory actions, osteogenic differentiation promotion, and angiogenic stimulation, collectively contributing to enhanced bone defect repair [59,60,61,62].

### 3.3. Loading Bioactive Ions into Scaffolds

Extensive research has demonstrated that various bioactive ions, including Mn^2+^, Sr^2+^, Ag^+^, Mg^2+^, Zn^2+^, and Cu^2+^, play crucial regulatory roles in the local immune microenvironment during bone regeneration. These ions exhibit multiple therapeutic effects such as antimicrobial activity, promotion of stem cell osteogenic differentiation, inhibition of osteoclast activity, modulation of macrophage polarization, and stimulation of vascular regeneration, all of which contribute to enhanced bone regeneration. Consequently, incorporating these bioactive ions into bone tissue engineering scaffolds enables localized biological regulation, demonstrating promising potential for promoting bone repair. Currently, bioactive ion-loaded bone scaffolds have emerged as a research focus in the field. Specific applications of these ion-loaded scaffolds are summarized in Table 1.

#### 3.3.1. Loading of Manganese Ions

Manganese ions (Mn^2+^) are an essential trace element that significantly influence bone metabolism and integrity. Current research demonstrates that Mn^2+^ actively participates in bone remodeling processes by modulating the activities of osteoblasts, osteoclasts, and other key cellular components involved in bone formation and resorption. Furthermore, Mn^2+^ affects bone mineralization through the regulation of complex cellular signaling pathways and enzymatic reactions [81].

Recent studies have revealed that incorporating Mn^2+^ into bone tissue engineering scaffolds enhances osteogenesis through multiple mechanisms: (1) the MnSOD/AMPK pathway-mediated recruitment of CD4^+^ T cells at bone defect sites; (2) promotion of Th2 polarization coupled with suppression of Th1 polarization, thereby creating an anti-inflammatory microenvironment conducive to bone regeneration [65]. Earlier studies have revealed that Mn^2+^-loaded scaffolds achieve sustained ion release, which significantly mitigates inflammatory responses by upregulating the M2 macrophage phenotype. This process concurrently activates the hypoxia-inducible factor-1α (HIF-1α) pathway to promote angiogenesis while inhibiting osteoclast formation and function, ultimately facilitating bone regeneration [63,64].

#### 3.3.2. Loading of Silver Ions

Extensive research has demonstrated that silver ions (Ag^+^) at appropriate concentrations exhibit potent antimicrobial activity. The antibacterial mechanisms involve: (1) penetration through porin proteins or formation of membrane pores leading to cellular membrane damage and cytoplasmic leakage; (2) induction of reactive oxygen species (ROS) generation; (3) protein denaturation and DNA damage [52]. Owing to these beneficial properties, silver nanoparticles (Ag NPs) and silver ions hold considerable value for incorporation into bone tissue scaffolds for the treatment of infected bone defects.

For instance, Mingjie Sun et al. [66] developed a nano-hydroxyapatite/poly(ethylene glycol) diacrylate (nHA/PEGDA) composite scaffold incorporating Ag NPs. This system demonstrated sustained silver ion release post-implantation, exhibiting broad-spectrum antibacterial effects while effectively promoting the healing of infected bone defects. Another approach consisted of a dissolution-precipitation method to load silver phosphate into a honeycomb-structured bone scaffold. This design enabled an immediate burst release of Ag^+^ upon implantation, followed by prolonged release over several months, achieving effective infection prevention and enhanced bone regeneration [67].

#### 3.3.3. Loading of Strontium Ions

Strontium (Sr) has been established as an osteoimmunomodulatory factor capable of promoting bone repair through dual mechanisms: suppression of osteoclast proliferation and enhancement of vascularization [68,69,70,82]. Strontium ranelate was approved in 2004 for the European market for osteoporosis treatment, highlighting its potential as a bioactive component in bone tissue engineering scaffolds.

A representative study by Qiuju Miao et al. [70] incorporated strontium ions (Sr^2+^) into porous 3D-printed tricalcium phosphate scaffolds. The released Sr^2+^ exhibited multifaceted therapeutic effects: (1) modulation of the local immune microenvironment by promoting macrophage polarization from the pro-inflammatory M1 phenotype to the anti-inflammatory M2 phenotype; (2) stimulation of M2 macrophages to secrete elevated concentrations of vascular endothelial growth factor (VEGF) and platelet-derived growth factor-bb (PDGF-bb). This cytokine cascade resulted in the formation of a dense, highly branched vascular network within the bone defect region, ultimately accelerating the rate of new bone formation.

#### 3.3.4. Loading of Magnesium Ions

Magnesium ions (Mg^2+^) possess unique capabilities to stimulate osteogenic differentiation and bone formation [83,84]. When incorporated into bone tissue engineering scaffolds, Mg-based materials demonstrate immunomodulatory effects by regulating macrophage polarization, reducing pro-inflammatory cytokine production, and rapidly resolving local inflammation while enhancing tissue repair capacity [71,73,85]. These properties position Mg-loaded scaffolds as promising candidates for bone replacement therapies. However, direct application of magnesium alloy scaffolds may result in excessively high local Mg^2+^ concentrations that adversely affect bone regeneration [86]. To address this, researchers have developed various Mg-containing bone scaffolds with controlled ion release profiles. For instance, innovative approaches utilizing piezoelectric sustained-release systems and Mg-loaded nanoparticles have successfully achieved controlled Mg^2+^ release. These systems promote bone regeneration through multiple mechanisms: (1) inducing M2 macrophage polarization; (2) inhibiting osteoclast activation; (3) enhancing osteogenic differentiation at bone defect sites [72,74].

#### 3.3.5. Loading of Zinc Ions

Zinc ions (Zn^2+^) represent an essential trace element for skeletal development and exhibit dose-dependent osteogenic and osteoclastic effects. Recent studies have shown that low Zn^2+^ concentrations promote osteoblast proliferation and differentiation via the Wnt/β-catenin pathway, while higher concentrations induce osteoclast differentiation through NF-κB signaling [76,77]. Researchers have harnessed this dual regulation by incorporating zinc submicron particles into scaffolds. Such designs enable long-term, stable release of therapeutic Zn^2+^ doses that enhance BMSC adhesion and osteogenic differentiation, resulting in superior osteogenic and anti-inflammatory properties [75].

#### 3.3.6. Loading of Copper Ions

Copper ions (Cu^2+^), recognized essential metal ions in human physiology, have been demonstrated to possess multifunctional therapeutic properties, including antimicrobial activity, angiogenic potential, and osteogenic effects [87,88,89]. Ying Yang et al. [80] developed a novel bone tissue scaffold by incorporating copper nanoparticles (CuNPs) into a photo-crosslinked graphene oxide-gelatin methacryloyl (GO-GelMA) hydrogel system. This construct achieved sustained Cu^2+^ release, which significantly enhanced osteogenic differentiation, M2 macrophage polarization, and anti-inflammatory cytokine secretion. In vivo evaluations confirmed the scaffold’s efficacy in promoting bone regeneration and accelerating healing in critical-sized calvarial defects [78,79].

### 3.4. Loading of Other Bioactive Components into Scaffolds

In addition to the aforementioned loading strategies, recent advances have explored incorporating mesenchymal stem cells (MSCs) and biologically active substances derived from human tissues or cells, such as platelet-rich plasma (PRP) and extracellular vesicles (EVs), into bone tissue engineering scaffolds. These approaches provide both cellular sources and abundant bioactive factors to the defect site, enabling precise biological modulation of the local microenvironment post-implantation, thereby promoting bone regeneration.

A representative study by Gan Wang et al. [90] demonstrated the synergistic potential of this strategy by co-loading adipose-derived stem cells (ADSCs) with PRP into 3D-printed porous titanium alloy scaffolds. The osteogenic and angiogenic factors in PRP significantly promoted ADSC proliferation, osteogenic differentiation, and intra-scaffold vascularization, with in vivo studies confirming superior bone defect repair efficacy. Emerging research has employed MSC-derived small extracellular vesicles (sEVs) in bone scaffolds, demonstrating that sustained sEV release promotes osteogenesis through three coordinated mechanisms: (1) induction of osteogenic differentiation; (2) stimulation of angiogenesis; (3) modulation of local inflammatory microenvironments [91,92,93,94,95].

Collectively, these advanced loading strategies enhance the bio-regulatory capacity of bone scaffolds. The diverse incorporated components exert their therapeutic effects through distinct yet complementary mechanisms to optimize the local microenvironment for bone regeneration. Substantial experimental evidence confirms that these approaches significantly enhance the osteogenic performance of tissue-engineered scaffolds, offering novel solutions for the clinical management of critical-sized craniofacial bone defects.

## 4. Summary and Future Perspectives

In summary, craniofacial bone defects have various etiologies and exhibit a high clinical incidence [96,97]. Nonetheless, traditional therapeutic approaches, such as autologous bone grafts, allogeneic bone grafts, or distraction osteogenesis, often yield suboptimal outcomes. Consequently, there is an urgent need to explore novel treatment strategies. Notably, artificial bone tissue engineering scaffolds have emerged as a promising alternative [98,99].

Early-stage bone tissue engineering scaffolds primarily focused on fundamental properties such as structural stability, mechanical strength, and biocompatibility [100,101,102]. However, with a deeper understanding of the complex mechanisms of bone regeneration, more sophisticated and functionally refined scaffolds have been developed, featuring enhanced bio-regulatory capabilities [103,104]. Loading strategies have proven to be an effective approach to achieve this goal.

Through techniques such as 3D printing, nanoencapsulation, chemical crosslinking, and physical doping, bone tissue engineering scaffolds can be loaded with cell regulatory factors, pharmaceuticals, bioactive ions, stem cells, or viable components derived from human tissues or cells [11,105,106]. These modifications enhance bio-regulatory functions of the scaffolds during bone regeneration by influencing key cellular processes, including macrophage polarization, osteoblast and osteoclast activity, and mesenchymal stem cell differentiation, as well as modulating cellular signaling pathways, local microbiological conditions, defect-site vascularization, bone tissue mineralization, and so on [107,108].

Researchers have developed various bone tissue engineering scaffolds with bio-regulatory capabilities through loading strategies, and their safety and osteogenic-promoting efficacy have been demonstrated in both in vitro and in vivo animal experiments. Despite the promising results outlined herein, it is crucial to critically appraise the current limitations. The field lacks standardized criteria for evaluating material biocompatibility and mechanical performance, making direct comparisons between studies challenging. Furthermore, the findings predominantly rely on small-animal models (rats, rabbits), which have limited translational relevance to human pathophysiology due to differences in bone size, healing rates, and immune system function [34,109]. The scarcity of large-animal studies and human clinical data for most advanced scaffolds represents a significant translational gap [110].

Nevertheless, several other challenges and unresolved issues remain to be addressed prior to clinical translation. These include but are not limited to: (1) refinement of spatiotemporal control over the release of loaded bioactive components; (2) long-term safety and efficacy evaluation of the bio-regulatory functions; (3) potential off-target effects and the stability, storage, and shelf life of bioactive molecules loaded into scaffolds; (4) standardization and scalability in the manufacturing of scaffolds employing loading strategies; (5) development of more advanced preclinical models.

Additionally, issues of long-term safety, potential off-target effects of growth factors, and the practical challenges associated with the storage and shelf-life of bioactive-laden scaffolds remain largely unexplored

In conclusion, loading strategies have revolutionized bone tissue engineering, endowing scaffolds with unprecedented bio-regulatory precision. While these innovative scaffolds demonstrate significant potential in pre-clinical settings for craniofacial bone regeneration, their clinical translation requires overcoming substantial hurdles. Future work must focus on standardizing evaluation methods, conducting robust large-animal and clinical studies, and meticulously addressing long-term safety and manufacturing stability. By integrating insights from immunology, materials science, assessment system, and clinical orthopedics, next-generation scaffolds employing advanced loading strategies show promising potential for the functional and aesthetic restoration of diverse craniofacial bone defects.

## Figures and Tables

**Table 1:** A summary of studies on bioactive ions-loaded bone tissue engineering scaffolds promoting bone regeneration.

Ions	Loading Strategy	Bioactive Effect	Related Signaling Pathway	Animal Model	Ref.
Mn^2+^	add the manganese carbonyl (MnCO) nanosheets to the biomimetically hierarchical scaffold	stimulate the M2 polarization of macrophages and alleviate inflammatory responses, enhance neovascularization,	the hypoxia-inducible factor-1α (HIF-1α) signaling pathway	Rat critical-size femur bone defect model	[63]
	use a coprecipitation method to add MnCl_2_ into the β-tricalcium phosphate (β-TCP) bioceramic scaffold	scavenge ROS, inhibits the formation and function of osteoclasts, enhance ALP activity and the mineralization of MC3T3-E1 cells	Reactive oxygen species-receptor activator of nuclear factor κB ligand (ROS-RANKL) signaling pathway	Rat OVX-related femoral defect regeneration model	[64]
	add the Mn-Si-hydroxyapatite nanowires to the gelatin methacryloyl scaffold	heighten recruitment of CD^4+^ T cells, escalate T helper (Th) 2 polarization, abate Th1 cell polarization, foster the osteogenesis of bone marrow stromal cells	MnSOD/AMPK signaling pathway	CD^4+^ T cell-depleted murine model, murine mandibular bone defect model	[65]
Ag^+^	use the in situ growth technique to embed Ag nanoparticles to the polydopamine-mesoporous silicon nanoparticle which were introduced into PLLA scaffolds through Selective laser sintering	have sterilizing effect on both Gram-negative *Escherichia coli* (*E. coli*) and Gram-positive *Staphylococcus aureus* (*S. aureus*)	Not mentioned	Not mentioned	[52]
	mix AgNPs and GO with nanohydroxyapatite/polyethylene glycol diacrylate (AGHP) to fabricate a 3D-printed composite scaffold	the hydrolyzed silver ions have an excellent killing performance against G^+^ and G^−^ bacteria	Not mentioned	Not mentioned	[66]
	Surface modification of carbonate apatite (CAp)—honeycomb scaffold with Ag_3_PO_4_	prevent bacterial adhesion to the scaffold, ehance the bone formation	the signaling pathways of integrin α5, transforming growth factor beta	Rabbit femur condyles bone defect model	[67]
Sr^2+^	directly mix SrCl_2_ and Alginate-Dopamine aqueous solutions to fabricate a multifunctional hydrogel scaffold	promote the mineralization and the osteogenic differentiation	Not mentioned	Rat skull defect model	[68]
	coating 3D PCL scaffold with carboxymethyl kappa-carrageenan, and then create strontium phosphate layer via a modified alternate soaking process	improve attachment and viability of the MSCs and accelerate osteogenic differentiation	Runx 2—OCN signaling pathway	Not mentioned	[69]
	dope strontium ions (Sr) into TCP (SrTCP) and prepare porous 3D printed Sr-TCP scaffolds by 3D printing	inhibit the expression of pro-inflammatory genes, promote the polarization of macrophages from M1 to M2	Not mentioned	Rabbit full-thickness tibial plateau bone defect model	[70]
Mg^2+^	use the digital light processing (DLP) method to fabricate β-TCP scaffolds doped with magnesium oxide with gyroid structure	promote the osteogenic differentiation of bone marrow mesenchymal stem cells (BMSCs), angiogenic differentiation and the polarization of macrophages from M1 to M2	Not mentioned	Rabbit femur condyles bone defect model	[71]
	fabricate a 3D printing composite scaffold composed of piezoelectric whitlockite (a natural magnesium-containing calcium phosphate) and poly(ε-caprolactone) (PCL)	Inhibit osteoclast activation, promote the neurogenic, angiogenic, and osteogenic differentiation of bone marrow mesenchymal stromal cells	Not mentioned	Rat calvarial defect model	[72]
	mix the silk fibroin Gly-solution and Laponite solution, pour into a mold and fabricated porous scaffold by lyophilization	facilitate stem cell osteogenic differentiation	TRPM 7-PI3K-AKT signaling pathway	Rat alveolar bone defect model	[73]
	constructe a bionic cancellous bone scaffolding system α/β-tricalcium phosphate (α/β-TCP) by low-temperature 3D printing, and gelatin is preserved inside the scaffolds, and later loaded with tea polyphenol-magnesium (TP-Mg) nanoparticles	promote the expression of osteogenic genes, inhibit S.aureus and promote the transition of macrophages from M1 to M2 phenotype	Runx 2—OCN signaling pathway	Rat infected skull defect model	[74]
Zn^2+^	add zinc submicron particles to PLGA/β-TCP using low temperature rapid prototyping 3D printing technology	promote the adhesion of BMSCs, enhance the osteoinductivity and promote the osteogenic activity and mineralization process of BMSCs	Wnt/β-catenin signaling pathway, P38 MAPK signaling pathway	Rat femoral condyle defect model	[75]
	fabricate PCL/Zn composite scaffolds with different Zn powder contents through fused deposition modelling	promote osteogenesis in a dose-dependent manner within a certain range, when it exceed this range, osteoclastogenesis is promoted;	Wnt/β-catenin signaling pathway, NF-κB signaling pathway	Rat skull defect model	[76]
	incorporate different amounts of ZnO microcrystalline bioactive glass to fabricate macroporous Zn-MCBG scaffolds	an appropriate concentration of Zn^2+^ could promote the polarization of macrophages from M1 to M2 and the osteogenic differentiation of BMSCs	Not mentioned	Rat skull defect model	[77]
Cu^2+^	add a calcium sulfate-Cu^2+^ delivery system to the stereolithography (SLA) 3D-printed calcium silicate artificial bone	exert a long-lasting antimicrobial effect, promote vascular growth, increase ALP activity and induce BMSC mineralization	Not mentioned	Rat skull defect model	[78]
	fabricate a Cu-doped composite scaffold of nano calcium-deficient hydroxyapatite (n-CDHA)/multi(amino acid) copolymer (MAC)	promote osteogenesis and angiogenesis	Not mentioned	Rabbit femur condyles bone defect model	[79]
	combine copper nanoparticle (CuNP)-decorated graphene oxide (GO) nanosheets (GO/Cu) with methacrylated gelatin (GelMA) to construct porous bone scaffolds	promote the recruitment of macrophage and M2 type macrophage polarization after injury, enhance the osteogenic differentiation of BMSCs, promote neovascular formation and infiltration	Not mentioned	Rat calvarial defect model	[80]

## Data Availability

No primary research results, software or code have been included and no new data were generated or analysed as part of this review.
